# Diagnostic accuracy of oral cancer cytology in a pilot study

**DOI:** 10.1186/s13000-017-0618-3

**Published:** 2017-03-16

**Authors:** Joji Sekine, Eiji Nakatani, Katsumi Hideshima, Teruaki Iwahashi, Hiroshi Sasaki

**Affiliations:** 1 Japanese Society of Clinical Cytology, Tokyo, Japan; 2 Department of Oral and Maxillofacial Surgery, Shimane University Faculty of Medicine, 89-1 Enya-cho, Izumo, Shimane 693-8501 Japan; 3 Translational Research Informatics Center, Foundation for Biomedical Research and Innovation, Kobe, Japan; 4 Department of Obstetrics and Gynecology, The Jikei University Kashiwa Hospital, Kashiwa, Japan

**Keywords:** Accuracy, Cytology, Oral cancer, Pathology, Diagnosis

## Abstract

**Background:**

Recently, cytology has been applied to the diagnosis of oral lesions. We aimed to explore the diagnostic accuracy of oral cytology based on the histological diagnosis.

**Methods:**

Histological diagnoses of 327 cases were classified as Negative, Borderline lesion –, Borderline lesion +, oral intraepithelial neoplasia/carcinoma in situ (OIN/CIS), or Positive. Cytological diagnoses were classified as NILM (negative for intraepithelial lesion or malignancy), LSIL (low-grade squamous intraepithelial lesion), HSIL (high-grade squamous intraepithelial lesion), or SCC (squamous cell carcinoma). The cytology slides were evaluated by 10 raters and the results were compared with the histology results.

**Results:**

In 142 cases that were histologically negative, the number of NILM, LSIL, HSIL, and SCC and other malignancy was 77 (54.2%), 47 (34.3%), 8 (5.6%), and 10 (7.0%), respectively. Among 32 cases of Borderline lesion –, the number of NILM, LSIL, HSIL, and SCC and other malignancy was 11 (34.3%), 11 (34.3%), 9 (28.1%), and 1 (3.1%), respectively. Also, in 4 cases of Borderline lesion +, the number of NILM, LSIL, HSIL, and SCC and other malignancy was 2 (50.0%), 0 (0.0%), 0 (0.0%), 2 (50.0%), respectively. Among 12 cases of OIN/CIS, the number of NILM, LSIL, HSIL, and SCC and other malignancy was 1 (8.3%), 2 (16.7%), 4 (33.3%), and 5 cases (41.7%), respectively. Among 137 cases with a histological diagnosis of Positive, the number of NILM, LSIL, HSIL, and SCC and other malignancy was 7 (5.1%), 22 (16.1%), 19 (13.9%), and 89 (65.0%), respectively. Sensitivity, specificity, and positive predictive and negative predictive values were 93.5, 50.6, 62.4, and 89.8%, respectively, when the cytological diagnosis of Negative was assumed to be NILM; they were 77.8, 83.9, 81.0 and 81.1%, respectively, if the cytological diagnosis of Negative was assumed to be NILM and LSIL. The number of false-positive and false-negative diagnosis affected cases with LSIL and HSIL may indicate the difficulty in the cytological diagnosis of borderline lesions. While the negative predictive value was relatively high (89.8%) when cytological Negative was assumed to be NILM only.

**Conclusion:**

Histopathological examination should be recommended in cases with cytological diagnoses of LSIL, HSIL, and SCC.

## Background

Exfoliative cytology is a reliable tool for assessing malignant change in various organs [1]. Cytology has been also applied to the diagnosis of oral lesions [2–4]. Accurate cytological diagnosis of oral lesions, especially in distinguishing benign lesions from malignant ones, is essential for treatment as well as for clinical and epidemiological research including the study of prognosis [5].

With respect to cytological diagnosis, the classification of cervicovaginal smears into five classes was initially proposed by Papanicolaou, who formulated a series of guidelines for smear interpretation [6]. This system was generally well received, although the significance of the classes was often modified to meet the requirements of laboratories in consultation with clinicians [7]. In December 1988, a committee of experts who convened under the auspices of the National Cancer Institute (USA) in Bethesda, Maryland, proposed a diagnostic system for the interpretation of cervicovaginal smears. The resulting Bethesda System (modified in 2001) was officially accepted by the federal authorities in the United States [8]. Recently, this system has been used in various fields such as thyroid, renal, and female genital cytology [9].

Fuller et al. pointed out that although oral cytology seems to have a higher diagnostic value, further study is needed to assess this [10]. However, the accuracy of oral cytology is unknown. Similar to other fields, diagnosis in oral cytology has been based on the Papanicolaou classification, not by Bethesda System [8, 9]. However, there had been no established diagnostic guideline in Japan. The Japanese Society of Clinical Cytology (JSCC) then organized a working group for oral cytology to confirm the accuracy of oral cytology according to Bethesda System [8, 9] in 2013, and established a diagnostic guideline committee for oral cytology in 2013.

This study investigated the clinical applicability of oral cytology following Bethesda System [8, 9] before the publication of diagnostic guideline by JSCC [11] by exploring the diagnostic accuracy of oral cytology based on histological diagnosis as the gold standard.

## Methods

### Samples

Patients diagnosed with oral disease were examined in this study. All cytology samples were prepared from benign or malignant oral lesions for biopsy and/or surgically resection. The samples were prepared by smearing collected cells on glass slides, which were then immersed in 95% ethanol, fixed, and stained with Papanicolaou staining. The samples derived from the patient population had results of oral cytology. Data from five Japanese institutions were included (Department of Oral and Maxillofacial Surgery, Shimane University Faculty of Medicine; Department of Oral Pathology, School/Graduate School of Dentistry Osaka University; Second Department of Oral and Maxillofacial Surgery, Osaka Dental University; Department of Health Promotion, Division of Oral Pathology, Kyushu Dental University and Department of Oral Pathology, Division of Oral Pathogenesis and Disease Control, Asahi University School of Dentistry). The samples (including data) were retrospectively collected in clinical practice between October 2007 and November 2013, and cytological and histological tests were performed simultaneously.

This study was planned and performed following STARD checklist for reporting of studies of diagnostic accuracy (http://www.stard-statement.org) (data not shown).

### Procedure of cytological diagnosis

All the raters who passed the board examination for cytology of JSCC reviewed the samples. The slides were evaluated independently by at least two raters, and a representative cytology result of each case was determined by a majority vote.

Cytological diagnoses were performed based on Bethesda System [8, 9], and were classified into NILM (negative for intraepithelial lesion or malignancy), LSIL (low-grade squamous intraepithelial lesion), HSIL (high-grade squamous intraepithelial lesion), SCC (squamous cell carcinoma), and IFN (indefinite for neoplasia or non-neoplasia) (Table 1).

### Procedure of histological diagnosis

A histological diagnosis was provided by oral pathologists at each institution, and then the number of biopsy samples was determined at the investigator’s discretion. These histological slides underwent hematoxylin and eosin staining. Their histological findings were classified into five categories as follows. Negative was defined as non-malignant lesions including inflammatory ones; Borderline lesion – was defined as mild, mild–moderate, or moderate dysplasia; Borderline lesion + was defined as severe dysplasia; OIN/CIS was defined as oral intraepithelial neoplasia or carcinoma in situ; and “Positive” was defined as squamous cell carcinoma and other malignancies. Borderline lesion – and Borderline lesion + were prepared based on general rules for clinical and pathological studies on oral cancer [11, 12].

Accordingly, the results of representative cytology were compared with the histology results.

### Statistical analysis

To compare between cytological and histological diagnosis, the histological diagnoses were classified into Negative (Negative and Borderline lesion –) and Positive (Borderline lesion +, OIN/CIS and Positive), and cytological diagnosis were also classified into Negative (NILM) and Positive (LSIL, HSIL, SCC, Other malignancy) or Negative (NILM and LSIL) and Positive (HSIL, SCC, Other malignancy). To examine the diagnostic performance by comparing their cytological diagnosis against the histological diagnosis, the sensitivity, specificity, positive predictive value, and negative predictive value were calculated, and all statistical analysis was performed using SAS version 9.3 (Cary, NC).

## Results

### Samples

A total of 423 samples of oral cytology with accompanying histological slides from five institutions were screened. Among the 423 samples, 96 samples (22.7%) were excluded from the study because of poor quality (e.g., scant cellularity or air-drying artifact). The remaining 327 samples were reviewed. The remaining 327 samples were reviewed by 10 raters. Among these samples, 93, 47, 82, 80, 18, 1, and 6 samples were reviewed by 2, 3, 4, 5, 6, 7, 8 raters, respectively (Table 2).

**Table 1:** Diagnostic guideline for oral cytology, proposed by the Diagnostic Guideline Committee for Oral Cytology of the Japanese Society of Clinical Cytology

Abbreviation	Corresponding pathological diagnosis
NILM	normal, infection, inflammation, lichen planus, leukoplakia, benign epithelial lesion, etc.
LSIL	mild and moderate dysplasia, and SIN1 and SIN2; mentioned in WHO 2005
HSIL	severe dysplasia, carcinoma in situ, and SIN3; mentioned in WHO 2005
SCC	squamous cell carcinoma
Other malig.	other malignancy
IFN	indefinite for neoplasia or non-neoplasia

Cytological diagnoses were classified as NILM (negative for intraepithelial lesion or malignancy), LSIL (low-grade squamous intraepithelial lesion), HSIL (high-grade squamous intraepithelial lesion), or SCC (squamous cell carcinoma) and IFN (indefinite for neoplasia or non-neoplasia)

**Table 2:** The numbers of reviewing samples per number of raters

Number of raters	Number of samples (%)
2	93 (28.4)
3	47 (14.4)
4	82 (25.1)
5	80 (24.5)
6	18 (5.5)
7	1 (0.3)
8	6 (1.8)

### Histological diagnosis

The histological diagnoses of 327 cases were classified as Negative, Borderline lesion –, Borderline lesion +, OIN/CIS, or Positive (Table 3).

**Table 3:** Histopathological categories of the reviewed samples

Details (*n* = 327)	Histopathological diagnosis				
	Negative		Positive		
	Negative (*n* = 142)	Borderline lesion – (*n* = 32)	Borderline lesion + (*n* = 4)	OIN/CIS (*n* = 12)	Positive (*n* = 137)
Benign tumor	41				
Inflammation	41				
Leukoplakia	21				
Lichen planus	14				
No malignancy	10				
Epulis	9				
Mucocele	4				
Candidiasis	1				
Pemphigus vulgaris	1				
Dysplasia mild		28			
mild–moderate		2			
moderate		2			
severe			4		
OIN/CIS				12	
Squamous cell carcinoma					130
Other malignancy					7

*OIN* oral intraepithelial neoplasia, *CIS* carcinoma in situ

### Result of cytological diagnosis compared with histological diagnosis

Table 4 shows the results for cytological diagnoses compared with histological diagnoses. In 142 cases that were histologically negative, the number of patients with NILM, LSIL, HSIL, and SCC and other malignancy was 77 (54.2%), 47 (34.3%), 8 (5.6%), and 10 (7.0%), respectively. Among the 32 cases of Borderline lesion –, the number of patients with NILM, LSIL, HSIL, and SCC and other malignancy was 11 (34.3%), 11 (34.3%), 9 (28.1%), and 1 (3.1%), respectively. Also, in the 4 cases of Borderline lesion +, the number of patients with NILM, LSIL, HSIL, and SCC and other malignancy was 2 (50.0%), 0 (0.0%), 0 (0.0%), 2 (50.0%), respectively. Among the 12 cases of OIN/CIS, the number of patients with NILM, LSIL, HSIL, and SCC and other malignancy was 1 (8.3%), 2 (16.7%), 4 (33.3%), and 5 cases (41.7%), respectively. Among 137 cases with a histological diagnosis of Positive, the number of patients with NILM, LSIL, HSIL, and SCC and other malignancy was 7 (5.1%), 22 (16.1%), 19 (13.9%), and 89 (65.0%), respectively.

**Table 4:** Results of cytological diagnoses compared with histopathological diagnoses

Cytological diagnosis *n* = 327	Histopathological diagnosis				
	Negative		Positive		
	Negative (*n* = 142)	Borderline lesion − (*n* = 32)	Borderline lesion + (*n* = 4)	OIN/CIS (*n* = 12)	Positive (*n* = 137)
NILM (*n* = 98)	77 (54.2%)	11 (34.3%)	2 (50.0%)	1 (8.3%)	7 (5.1%)
LSIL (*n* = 82)	47 (34.3%)	11 (34.3%)	0 (0.0%)	2 (16.7%)	22 (16.1%)
HSIL (*n* = 40)	8 (5.6%)	9 (28.1%)	0 (0.0%)	4 (33.3%)	19 (13.9%)
SCC (*n* = 104)	9 (6.3%)	1 (3.1%)	2 (50.0%)	5 (41.7%)	87 (63.5%)
Other malignancy (*n* = 3)	1 (0.7%)	0 (0.0%)	0 (0.0%)	0 (0.0%)	2 (1.5%)

*NILM* negative for intraepithelial lesion or malignancy, *LSIL* low-grade squamous intraepithelial lesion, *HSIL* high-grade squamous intraepithelial lesion, *SCC* squamous cell carcinoma, *OIN* oral intraepithelial neoplasia, *CIS* carcinoma in situ

### Distribution of histological diagnoses from the viewpoint of cytological diagnosis

**Fig. 1:**
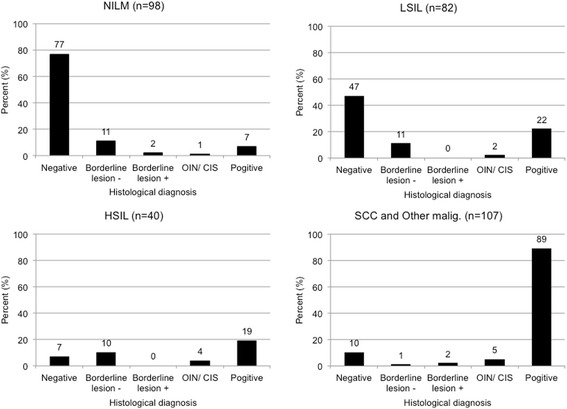
Distribution of the 327 cases with a histological diagnosis in each cytological diagnostic category. The percentages are shown above the columns

Fig. 1 shows the distribution of histological diagnoses from the viewpoint of cytological diagnosis. Among cases with a cytological diagnosis of NILM (98 cases), 78.6, 11.2, 2.0, 1.0, and 7.1% had a histological diagnosis of Negative, Borderline lesion –, Borderline lesion +, OIN/CIS, and Positive, respectively. Among cases with a cytological diagnosis of LSIL (82 cases), 57.3, 13.4, 0.0, 2.4, and 26.8% had a histological diagnosis of Negative, Borderline lesion –, Borderline lesion +, OIN/CIS, and Positive, respectively.

On the other hand, among cases with a cytological diagnosis of HSIL (40 cases) 20.0, 22.5, 0.0, 10.0, and 47.5% had a histological diagnosis of Negative, Borderline lesion –, Borderline lesion +, OIN/CIS, and Positive, respectively. Among cases with cytological diagnoses of SCC or Other malignancy (107 cases), 9.3 , 0.9, 1.9, 4.7, and 83.2% had a histological diagnosis of Negative, Borderline lesion –, Borderline lesion +, OIN/CIS, and Positive, respectively.

### Diagnostic performance of cytological diagnoses

With respect to cytological diagnostic performance, the sensitivity, specificity, positive predictive value, were 93.5, 50.6, 62.4, and 89.8% when cytological Negative was assumed to be NILM only, respectively (Table 5). Also, these measures were 77.8, 83.9, 81.0, and 81.1% when cytological Negative was assumed to be NILM and LSIL (Table 5).

**Table 5:** Diagnostic performance of cytological diagnoses

Discrimination of result of cytological diagnosis	Histopathological diagnosis^a^		Sensitivity (%)	Specificity (%)	Positive Predictive value (%)	Negative Predictive value (%)
	Positive (*n* = 153)	Negative (*n* = 174)				
Worse than NILM	143	86	93.5	50.6	62.4	89.8
NILM	10	88				
Worse than LSIL	119	28	77.8	83.9	81.0	81.1
NILM or LSIL	34	146				

*NILM* negative for intraepithelial lesion or malignancy, *LSIL* low-grade squamous intraepithelial lesion, *HSIL* high-grade squamous intraepithelial lesion, *SCC* squamous cell carcinoma, *OIN* oral intraepithelial neoplasia, CIS carcinoma in situ, ^a^: Positive included Positive, OIN/CIS, and Borderline lesion + based on the result of histopathological diagnosis, and Negative included Negative and Borderline lesion –

## Discussion

The cytological collection had various methods and instruments. In this study, all samples were conventionally smeared and Papanicolaou stained, although recently liquid-based cytology has become common in the field of gynecology [13, 14]. The conventional method is still common in the field of oral and maxillofacial surgery, despite the technical error of delay in fixation of the smear sample leads to an air-drying artifact [4]. Actually, in this study, 96 slides (22.7%) were deemed inadequate for review due to scant cellularity or air-drying artifact, all of which were prepared in one institution. Also, these inadequate samples were partially due to the fact that cytology instruments do not obtain a sample from the lesion [4], because the cytological instruments varied from cotton swabs to intratooth brush in period of sample collection. To improve the quality of the cytology slides, the methods and instruments of cytological collection should be reconsidered and standardized.

As most of oral neoplasms originated in squamous cell epithelium, the squamous epithelium should be deeply reviewed for accurate histological diagnosis of oral lesions [15]. In the field of oral histopahology, there has been controversy for classification or severity of oral epithelial dysplasia (OED) into categories of Borderline lesions + or – [12]. According to a literature [16], mild epithelial dysplasia refers to alteration in limited to the basal and parabasal layers, moderate epithelial dysplasia refers to alteration in the basal to mid-portion of the spsinous layer, and severe epithelial dysplasia refers to alteration that affect more than half of the thickness of the epithelium.

Because most dyslpastic changes occur in the basal and parabasal layers [17], to obtain a sample from the deepest layer of lesion is important to make an accurate cytological diagnosis of oral lesions derived from squamous cells. Further, because most cases of oral SCC are the differentiated type and are different from cervical SCC, most of the superficial cells of these lesions are not malignant and could be a reason of a false-negative diagnosis. An another reason for these “misdiagnoses” can be that the nuclear morphology of oral epithelial cells is easily affected by malignant transformation as well as inflammation and oral bacilli, so the shape of the nuclei would seem to be atypical and dysplastic, which would make accurate diagnosis in oral cytology difficult [14].

The uncertain cytological diagnosis of borderline lesions may affect our results as shown in Figure 1. However, in previous study [4, 18–20], detail of diagnoses in borderline lesions was not available. In future, the detailed information for borderline lesions should be investigated regarding the oral cytological diagnoses.

As borderline lesions diagnosed as LSIL and HSIL are then indicated for surgical resection in our hospital [5, 21], we strongly recommend that cases with cytological diagnoses of LSIL, HSIL SCC and Other malignancy should belong to the category of cytological positive. In our study, the number of false-positive and false-negative diagnosis affected cases with LSIL and HSIL may indicate the difficulty in the cytological diagnosis of borderline lesions (Table 4, Fig. 1). While the negative predictive value was relatively high (89.8%, Table 5) when cytological Negative was assumed to be NILM only.

As with most white oral lesions, the color is derived from the thickened keratin layer or thickened spinous layer, which masks the vascularity (redness) of the underlying connective tissue [16]. Accurate diagnosis of such white lesions is clinically difficult [16, 17], and a precise diagnosis of dysplasia in intraepithelial lesions is difficult even in histopathologic specimens [16]. Recently, Sekine et al [21] reported that nucleus accumbens-associated protein 1 (NAC1) has the potential to be used as a biomarker for distinguishing OED from CIS/OSCC. Standardization of the diagnosis of borderline lesions such as epithelial dysplasia is needed from a cytopathological viewpoint. There are very few studies on the accuracy of oral cytology [22], so it still remains unknown whether our results are satisfactory.

In this study, the cytology slides were evaluated by 10 raters and the results were compared with the histology results. Though each rater evaluated certain samples only one time in this study, one limitation was that the intra-examiner reliability was not able to be evaluated. In future, however, we will design and perform the further study to evaluate intra-examiner reliability on cytological diagnosis for oral cancer. Furthermore, revision of diagnostic guideline by JSCC [11] should be needed, as detailed classification, such as atypical squamous cells of undetermined significance (ASC-US) and atypical squamous cells cannot exclude HSIL (ASC-H) in the field of gynecology, should be considered to achieve more accurate diagnosis for borderline lesions in oral cytology.

## Conclusion

In conclusion, histopathological examination should be recommended in cases with cytological diagnoses of LSIL, HSIL, and SCCC.

## Abbreviations

ASC-H: Atypical squamous cells cannot exclude hsil
ASC-US: Atypical squamous cells of undetermined significance
CIN: Cervical intraepithelial neoplasia
CIS: Carcinoma in situ
HSIL: High-grade squamous intraepithelial lesion
IFN: Indefinite for neoplasia or non-neoplasia
JSCC: The Japanese society of clinical cytology
LSIL: Low-grade squamous intraepithelial lesion
NAC1: Nucleus accumbens-associated protein 1
NILM: Negative for intraepithelial lesion or malignancy
OED: Oral epithelial dysplasia
OIN: Oral intraepithelial neoplasia
SCC: Squamous cell carcinoma
